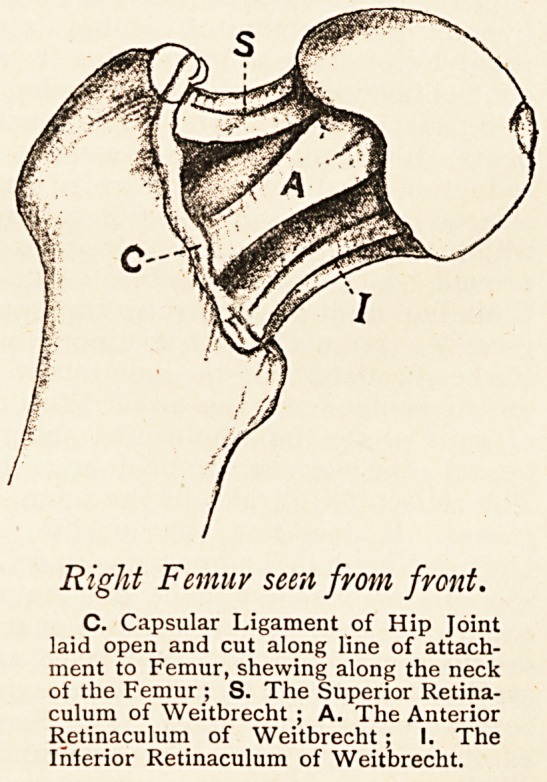# Meetings of Societies

**Published:** 1896-03

**Authors:** 


### MEETINGS OF SOCIETIES.

### Bristol Medico-Chirurgical Society.

*December 11th*, 1895.

Mr. Arthur W. Prichard, President, in the Chair.

Dr. F. H. Edgeworth showed a case of paralysis of the left external popliteal nerve in a middle-aged man, causing total paralysis of movement, with wasting and degenerative reaction, in the muscles innervated, and a partial anæsthesia of the skin area supplied. It was held that the interruption in the conductivity of the nerve fibres was probably due to a neuritis, analogous in etiology and pathology to that which takes place in peripheral facial paralysis.

Dr. A. B. Prowse showed specimens from a case of acute nephritis in a girl, aged 13. The attack, fatal at the end of the second week, followed a "chill;" and there was no history of scarlatina, or other preceding febrile condition. The œdema was at first excessive, but diminished considerably after treatment with jaborandi, digitalis, and hot-air baths. The urine quickly increased from 15 to 46 ounces *per diem*, but contained from .6 per cent. to 3 per cent. of albumen. During the last five days of life the motions were very watery and frequent. Two days before death the œdema of the legs increased so much that punctures were necessary to relieve the painful distension of the skin. The pulse failed very markedly about twelve hours before death, which occurred suddenly, after temporary improvement. At the necropsy the kidneys proved to be most typical specimens of the early large-white stage. The inferior vena cava was filled by a thrombus with a small central channel, and there were similar thrombi in all the large branches of the pulmonary artery. The vessels in the kidneys, also, were plugged more or less completely by decolorised clot.

Dr. Watson Williams read a paper on the "Symptoms and Diagnosis of Malignant Disease of the Larynx," referring especially to the slighter and less recognised early manifestations of malignant disease in this region. The great importance of early diagnosis of intrinsic laryngeal cancer lay in the fact that Semon's results proved that these cases occurring in patients in otherwise fair health, had a chance of 60 per cent. in favour of successfully undergoing radical surgical treatment, and of 50 per cent. of permanent relief from their disease, with a more or less useful voice. Thus the diagnosis of intrinsic cancer of the larynx was no longer always "a sentence of death," yet only too often cases became hopeless from failure to recognise the "danger signals" in laryngeal disease, so that the patient might come under the notice of an expert. The symptoms varied of course with the location of the new growth, but hoarseness was the commonest early symptom, not pain; pain, in fact, being often late in onset, or absent to the end. Glandular infiltration, too, was often insignificant or absent in early cases. Objectively, too, the early appearances were very deceptive. Cancer of the vocal cords might commence as a diffuse infiltration, with uneven surface, or merely as a congestion. In other cases a warty growth appeared, or a fringe-like outgrowth. Especially in the ventricular bands and aryteno-epiglottic folds, malignant growths were apt to commence as a diffuse infiltration, while epiglottic growths were more greyish-white in aspect. Hoarseness coming on in patients past middle life, and without obvious cause or general symptoms pointing to phthisis, syphilis, or gout, especially if associated with congestion of one cord or ventricular band, and particularly when the vocal cord movements were impaired, a symptom on which Semon laid much stress, was to be viewed with suspicion and carefully watched; the diagnosis having to be made from inflammatory disease, laryngeal paralysis, syphilis, tuberculosis, lupus, gout, pachydermia laryngis, benign growths, and perichondritis. Too much weight should not be attached to negative results of histological investigations of portions of growth removed, especially when clinical evidence pointed to malignancy; for a growth may present a papillomatous and benign surface with a malignant base.

Dr. Markham Skerritt showed a specimen of diffuse cancer of the stomach, in which practically the whole of the organ was infiltrated with the new growth, the thickness of its wall having an average of about an inch. The naked-eye appearance was typically that of colloid cancer. The case was of clinical interest chiefly on account of the resemblance which the growth in the stomach and adjacent parts had to a cancerous liver. The patient was a woman, aged 29, with a history of constitutional syphilis.—Dr. Theodore Fisher referred to the fact that although the growth was diffused through the stomach, very little contraction had taken place. As to the differentiation of such a growth from one in the pancreas or great omentum, he mentioned a case of diffuse carcinoma of the stomach that had come under his observation, in which he made a correct diagnosis from the fact that the mass hardened under palpation, showing it to contain muscular tissue.

Dr. Michell Clarke showed a specimen of a varicose aneurysm of the ascending aorta opening into the vena cava below the azygos vein by an aperture the size of a shilling. The right innominate vein was closed by a thrombus. The patient was an agricultural labourer, aged 53, who had enjoyed good health except for an attack of bronchitis the previous winter. Symptoms of intense dyspnœa, cyanosis, and swelling of the right upper extremity and of the face came on suddenly a month previous to observation. A loud musical double murmur was present at the base of the heart, which at times was continuous through systole and diastole.—Professor Fawcett called attention to the existence of a well-marked moderator band which connected the anterior musculus papillaris of the *left* ventricle with the septal wall. This band had evidently arisen by the dilatation of the ventricle pulling out an original trabecula carneæ which had in the pre-pathological state connected this anterior musculus papillaris with the septal wall. So attenuated had it become that it was, save for the part where it was connected with the base of the musculus papillaris, entirely tendinous.

Professor Fawcett gave a demonstration of the Retinacula of Weitbrecht, on which a paper by him was published in the *Journal of Anatomy and Physiology* for October, 1895. The neck of the femur is naturally a weak spot, because it is set on to the shaft at an angle and because it has to bear the weight of the body above it. During adult life it is strong enough for all emergencies, but in childhood and in old age the circumstances are very different. During childhood the head of the femur is united with the neck by a plate of cartilage, which is necessarily a source of weakness. In old age the cancellous and compact tissues of the neck of the femur become thinner and cause the neck to be consequently weaker. It is at these periods of life that the additional support afforded by these retinacula is most required. It is unlikely (1) that the periosteum surrounding the neck of the femur would be thickened along definite and constant lines if there were no cause for such thickening, or (2) that these bands exist for the mere purpose of anatomical demonstration or for the purpose of repair of fracture. As in the child the body-weight falls vertically over the head of the femur, we should expect to find two bands to prevent the head being displaced downwards, and we should not expect to find an anterior band because there would be nothing for it to do. An examination of the neck of a child's femur shows that such expectations are entirely realised. There are two bands, and two only, and they are in the positions we expect them to be—namely, above and

*Anterior view of neck of right Femur at birth.*

Capsule cut away to line of attachment to Femur, Retinacula of Weitbrecht exposed. **C.** Capsular Ligament. **S.** Superior Retinaculum of Weitbrecht. **I.** Inferior Retinaculum of Weitbrecht.

*Right Femur seen from front.*

**C.** Capsular Ligament of Hip Joint laid open and cut along line of attachment to Femur, shewing along the neck of the Femur; **S.** The Superior Retinaculum of Weitbrecht; **A.** The Anterior Retinaculum of Weitbrecht; **I.** The Inferior Retinaculum of Weitbrecht.

below. These upper and lower bands are evidently there by heredity —there to be ready to meet the strain they will soon be called upon to bear, there to prevent the head being torn from the neck of the femur,

—two of them only, because they have to meet at first with a vertical strain. But when once the erect condition is naturally attained, and the concomitant eversion of the limb bringing about the forward direction of the head and neck of the femur is established, the anterior part of the capsular ligament of the hip-joint and the psoas magnus having in the meantime, through repeated efforts at attainment and maintenance of the erect position, become lengthened out, a new strain is brought to bear on the epiphyseo-diaphyseal joint between the head and neck of the femur—a strain which is directed downwards and forwards from above and behind, and this strain has to be met by another band. That band is the middle or anterior retinaculum of Weitbrecht as we see it in the youth and adult. We may take it, then, that the retinacula of Weitbrecht are the strengthening fibres of the capsular ligament (cervical ligament of Stanley) of the joint between the head and neck of the femur, and that they are called forth by strains exerted in certain directions, and they limit excessive demands of those strains, preserving, by doing so, the integrity of the region involved; that the cervical ligament of Stanley being periosteum, fulfils the ordinary functions of periosteum—viz., those of repair and of nutrition; that the true function of the retinacula is prevention, not cure.

---

*January 8th*, 1896.

Mr. Arthur W. Prichard, President, in the Chair.

Mr. John Dacre read a paper as the introduction to a discussion on "The Use of Alcohol from a Medical Standpoint." (*See* page 10.)

Dr. Long Fox said that Sir Benjamin Richardson's experiments by hypodermic injection of alcohol showed that locally it acts as a neuro-paralytic. Taken internally, it removes in great measure the controlling influence of the smaller arteries on the heart, and causes also paresis of the vagus. The result is increase in number of cardiac beats, dilatation of surface-vessels, a feeling of surface-warmth, with reduction of the temperature of the body. It can scarcely be considered a food, as in itself it contains no one of the constituents of which the body is made. It gives no warmth to the body. This is proved by the thermometer. The disuse of it by deer-stalkers, by Canadian hunters, by Arctic explorers was additional proof of this. It gives no strength. It distinctly weakens the muscles. Professor Parkes realized this by experiment in the last Ashanti War. Acting on the same views, the Great Western Railway, during the alteration of rails along the whole line, substituted oatmeal gruel for alcohol, because the work had to be done with rapidity and with unusual energy. The relief of Chitral tells the same story, and so do our great national games. It does not lengthen life. To quote from the *Times*: "The United Kingdom Temperance and General Provident Society has two sections of insurers: one abstainers, the other alcohol users. An experience over 29 years shows that in the teetotal section, while 6187 deaths were expected, only 4368 took place; but in the section of alcohol users 8836 deaths were expected, and so large a number as 8017 actually took place. The Sceptre Life Assurance Society, the Scottish Temperance Life Assurance Company, and the Rechabites, all shew similar differences between these two classes of picked lives. The very lowest estimation of longer life is nine years on the teetotal insurers over those who are moderate drinkers." In many trades alcohol interferes with steadiness of hand, accuracy, and skill, and that where alcohol is taken in great moderation. Kraepelin's experiments shew that alcohol renders a longer time necessary for simple reaction, discrimination and selection with reference to his test by coloured discs. It blunts the judgment in testing weight: and even small doses apparently blunt in some persons the keenness of vision and the refined sense of hearing. It has been tersely stated that, compared with total abstinence, the moderate use of alcohol means for the generality of mankind shorter life, less work, worse work. Advice therefore given by the medical profession against the use of alcohol would be the highest kind of preventive medicine. Speaking generally, is there any one disease of the nervous system in which alcohol is advantageous? Dr. Fox gave it up in the treatment of *delirium tremens* 35 years ago, and with decided benefit to such cases. Judging from published accounts, success in the treatment of insanity is in direct ratio with the discontinuance of alcohol in any form. Is it useful in pneumonia or in fever? In both cases it is given with the idea of preserving life until defervescence occurs. It is probable that in all cases, especially in the young, the assistance given to the heart by the digitalis series is far more valuable than that by alcohol. Cases occur in which a few hours are of the greatest consequence: and it may be that the relief of a failing heart, even at the expense of partially paralysed arterioles, may occasionally help a patient over an important epoch. But such cases are exceptional. In old people, with diseased vessels and feeble digestion, a small quantity of alcohol may by dilating the arterioles of the stomach, induce a somewhat less feeble power of digestion; but if so, the effect would be a transient one. But for diseases of mucous surfaces, for all morbid affections of the liver, kidneys, or spleen, for every affection of the nervous centres and the nerves themselves, for tubercle and cancer with their neighbouring arterioles already diseased, as well as in the inroads of other micro-organisms as diphtheria *et hoc genus omne*, and for all morbid affections of the vascular system, the effect of alcohol as a medicine—that is, in moderate doses—is to increase the paralytic action, that is more or less a prominent feature in all morbid conditions. And it is poor physiology in the present day to advise its use to nursing mothers, with the view of increasing the secretion of milk.

Dr. A. J. Harrison said that the term "use" of alcohol is a very important one. In some cases—and crises of cases—alcohol is one of the most important therapeutic agents we possess, and is often so paramount as to excel all other modes of treatment. He was old enough to remember when to recommend alcohol to young children would bring upon one the censure of reprobation. Yet, at the same time, a few cases of excitable children were related, shewing great benefit from the exhibition of small doses—sleep and greater mental steadiness being produced. Delirium in pneumonia and fevers especially are indications for stimulants, and their exhibition is frequently followed by most satisfactory results. It is very good to give a small dose of brandy before an anæsthetic is used. It steadies the heart, and causes deeper inspirations to be made, and so more of the anæsthetic is inhaled in a shorter time. The late Dr. Anstie came to the conclusion that 2 oz. of absolute alcohol was the most that an adult should take in twenty-four hours. All wines and spirits are acid, only varying in degree of acidity. A small quantity of beer in many ladies with feeble digestion seems to be very beneficial indeed; food is then taken and enjoyed.

Mr. Munro Smith described a simple experiment carried out at the Medical School. Four beakers, each containing a definite amount of coagulated albumen and a digestive fluid, were placed in a warm chamber. To one beaker was added some port, to another sherry, to a third claret, and the fourth was left without alcohol. The result was this:

| | | |
|---|---|---|
| In the beaker without alcohol | ... | 2 grms were digested. |
| ,, with claret | ... | 1 grm. was ,, |
| ,, ,, sherry | ... | ·75 ,, ,, ,, |
| ,, ,, port | ... | ·65 ,, ,, ,, |

This shows the effect of different percentages of alcohol on *artificial* digestion; but in the living stomach the process is highly complex, and many other factors have to be considered, *e.g.* the vascularity of the organ, the churning movements and the secretion of HCl.; all these appear to be *increased* by the ingestion of alcohol. On the nervous system the effect of alcohol is clearly not entirely in the direction of paralysis. Physiological experiments show that there is an increased *vascularity* which counteracts or even overpowers the depressing effect; and there is evidence that the brilliant wits of the 18th century were at their greatest mental activity after moderate wine-drinking. The whole question is difficult, and dogmatic assertion on it should be condemned.

Dr. Shingleton Smith commented on the antiquity of the use of alcohol, and thought that a drug which had been so largely and increasingly in use since the times of Noah and of Job had obtained a venerable stability, which doubtless rested upon an excellent foundation. He thought that the physiological variations in the effects of the use of alcohol had not, perhaps, received the attention they deserved; the exaggerated sensibility of the tissues of young people, and the abnormal tendency to fibroid degeneration of various viscera, and more especially the tendency to degenerations of the nerve centres and the peripheral nerves varying much in different individuals, rendered it necessary that in such persons the amount of alcohol should be rigidly limited, and he considered that these pathological conditions were not always evidence of what is commonly called excess. He thoroughly believed in the utility of alcohol as a food, both in health and in disease, and that a moderate use of it in dietetics added largely to the happiness and welfare of mankind.

Dr. Markham Skerritt thought that with regard to the use of alcohol as a beverage in health, the discussion was tending to take up a retrograde and unscientific direction. For it was surely proved beyond dispute that healthy persons did not require alcohol, and in all circumstances of trial were better without it; and that the only question with them was whether they could take it with impunity because they liked it. The main conditions in which alcohol was useful in disease were: (1) Fevers, with an adynamic tendency. Here the amount that might often be taken with advantage was very remarkable; (2) States of weakness, as for instance in convalescence from acute disease, or in cases of cardiac weakness; (3) Some cases of dyspepsia, where a little alcoholic drink gave a stimulus to digestion; (4) Sometimes, conditions of persistent ill-health in children. The argument derived from the antiquity of alcohol and the Biblical references to it was amusing. It was quite plain that in the old days its reputation was very much what it was now: when the people heard men talking what sounded to them like nonsense, they immediately concluded that they were "full of new wine."

Mr. F. R. Cross considered that the moderate use of alcohol was desirable. He insisted upon the importance of using only the best forms, and denounced the practice of drinking cheap wines and spirits.

Dr. Arthur B. Prowse said that in regard to the action of alcohol on the heart, it was absolutely certain that in almost all cases, in health and in disease, the *frequency* of the beats was much increased; but that the evidence on which was based the assertion that it added to the *strength* of the beats was somewhat equivocal. No doubt there was an apparent improvement in this respect; but since the supposed test of this was usually the increased *volume* of the pulse, this is better explained by the undoubted effect of the drug in causing relaxation of the arterial system; which also allowed of the more rapid and easy transference of the blood into the veins. Consequently the pulse is short and soft. But the increase in the rate of beat is inimical to the welfare of the patient, because the diastolic period is appreciably shortened,—giving rise to impaired nutrition, and gradual exhaustion of the cardiac muscle. It is well known that a weak heart, especially in febrile states, tends to be a rapidly-acting one; and we all recognise the immense value which digitalis has, in suitable cases, by slowing the rate of beat, and by lengthening the diastole—thus favouring the nutrition of the muscular tissue, and its power of contraction. While he was Resident Medical Officer at the London Fever Hospital, Sir W. H. Broadbent had told him of an extended trial of the plan of treating *all* the cases admitted, the severe as well as the mild, without alcohol, which had been carried out there some years previously. The result was to show that the cases, with very few exceptions, got on as well without the use of stimulants, as under the then common plan of free stimulation by alcohol. Had time permitted, Dr. Prowse intended to have given some details of two instructive cases, which added to the evidence there is that, even in these days of so-called "Moderation," alcohol is frequently prescribed in toxic doses, which themselves give rise to unfavourable symptoms, — thoughtlessly attributed to the *disease*, instead of to the *drug*. The remark by Dr. Shingleton Smith to the effect that the use of alcohol has "added largely to the happiness of mankind," Dr. Prowse characterised as a most astounding statement; seeing that there is a very general recognition of the fact that the evidence (occasionally denied but never controverted) proves that the use of alcohol has led to an immense amount of misery, disease, and crime. He had come to the deliberate conclusion that it was the duty of medical men to discourage the use of alcohol in health; and to avoid its use in the treatment of disease whenever it was possible to substitute other drugs for it. His personal experience was that this substitution was almost always possible. Was it reasonable to go on freely using a drug which, as Dr. Dickinson had said, was the very "genius of degeneration"?

Dr. A. Harvey said that although the discussion had treated the question of the use of alcohol both from the therapeutic and dietetic sides, sufficient importance had not been attached to its use in pneumonia, and especially in the lobular pneumonia and capillary bronchitis of children: this is largely due to the action described by Dr. Long Fox of paralysis of the arterioles; the patient is bled into his own arterioles, and so a hampered right heart is unloaded. As a dietetic it is used rather as a sedative than as a stimulant, and the argument that it is not a stimulant is beside the question. The craving for sedatives will be gratified, and if alcohol is debarred, recourse will be had to worse drugs, as morphia and cocaine. Most men will agree with Omár Khayyám:—

> ' That much as wine has played the infidel
> And robbed me of my robe of honour, well
> I often wonder what the vintners buy
> That 's half as precious as the stuff they sell."

Mr. Greig Smith, who had not been present at the opening of the discussion, rose specially to express dissent from some of the views uttered by Dr. Prowse. He hoped that the meeting would be regarded as one of scientists, swayed by no social fads, but simply seeking all through Nature for what would best help them to save life and cure disease. On the face of it, if we desired an honest statement as to the values of alcohol, or opium, or meat, we should not go to teetotallers, or anti-opiumites, or vegetarians. They had prejudged the case from their own points of view, which ought not to be ours. If he thought that a patient of his would die for want of opium, or alcohol, or meat, that patient should be kept alive regardless of consequences from excess in these things. And that he hoped was the feeling of every man practising medicine. To keep a patient from alcohol for fear that he might become a drunkard, or from opium for fear that he might become an opium-eater, when alcohol or opium might have saved the patient's life, was, in his opinion, contrary to the true spirit of medicine. Further, abstention was a question of conscience and morality on the patient's part; the surgeon or physician used the best remedy he had, and if thereby he saved life his responsibility ceased. As a matter of fact patients treated freely by any drug during an acute illness were usually nauseated by that drug; this was a fortunate fact, but no real argument. If the patient's life was saved at the expense of his becoming an alcoholic or opium *habitué*, still he thought the result worth the risk. Dr. Shingleton Smith had stated that, on the whole, he believed that the human race were the happier if not the better for wine. Dr. Prowse had expressed his emphatic disapproval of this statement. Mr. Greig Smith heartily endorsed the opinion of Dr. Shingleton Smith, and made varions quotations from poets in support of his belief. He did not believe that drink was a very prolific cause of crime. The criminal was not a drunkard, his art did not permit of drunkenness; and the drunkard was not a criminal. The drunkard was an idle, stupid or careless ne'er-do-weel who might beat his wife (who may well have deserved it), but his brains were not equal to real crime. If he had a tendency to criminality, drunkenness would render him incapable of putting it into practice. In his practice Mr. Greig Smith gave less alcohol, and more, than most men. In ordinary cases he gave none. In bad cases he gave simply as much as the patient could absorb, and if the stomach were incapable of absorbing he gave it by the rectum. And in convalescence, particularly if prolonged, he knew nothing more valuable than really good Burgundy or port. Statistics of mortality regarding total abstainers and alcohol-drinkers were absolutely valueless, for they ignored other essential elements. They included children: that was a most disturbing factor. They took cognisance of the atrophic, perennial and uninteresting individual who wore the blue ribbon, and of the miserable, diseased creature who, while not a drunkard, tried to drown his cares and diseases in alcohol. Neither are drinkers, but the one dies before the other. And thus, if duration of life is to be the criterion of worthy manhood, and alcohol is to be the gauge, the teetotallers have it. The centenarian paupers (and of these there are many) are then to be encouraged at the expense of our early-dying Shelleys and Burnses (of whom there are few). To save life, to abjure humbug, to promote liberal and humane existence were, in Mr. Greig Smith's opinion, more important than to become abstainers from alcohol, to subscribe to creeds, and to pervert science. He believed alcohol to be one of the most valuable drugs in the Pharmacopœia, and he considered wine to be a real influence for good in living and thinking and ordinary work. Both in medicine and in ordinary life wine, like most other things, was occasionally abused. *Ex abusu non arguitur ad usum.*

Mr. John Ewens, referring to Mr. Greig Smith's observation that he was not responsible for the after-abuse of alcoholic drinks because he had to administer, in extreme cases of abdominal or other operations, large quantities of brandy, said he had never seen any such results, but rather a distaste was created by its free use under such circumstances. Mr. Ewens protested against the injudicious administration of wine, etc., to young women for attacks of depression, or low spirits, as he had seen many cases of intemperance induced thereby in after-life. He also objected to administer alcohol in any form to reclaimed drunkards when ill, as it was pretty certain to excite the old craving for it again. He believed that, generally speaking, the best, most lasting, and useful mental work was done by men who were total abstainers.

Dr. Aust Lawrence drew attention to the fact that in women between 35 and 45 years of age profuse menorrhagia was often caused by the immoderate use of stimulants. Excessive use of alcohol often was the cause of sterility in women otherwise perfectly healthy. Gin and water should not be given as a remedy for the pain at menstruation. In such cases a doctor should always be consulted. As an aid to lactation, the moderate use of stimulants to which a woman may be accustomed is of benefit; but it is no use to give her an extra amount with the idea that this will increase the amount of milk. In cases of septic *post-partum* troubles the free use of stimulants was always of very great service; as much as 10 to 20 oz. of brandy should be given in twenty-four hours.

Mr. Dacre, in reply, first commented upon the remarks of Dr. Fox, and with all deference begged to differ from him in principle. He considered that the statistics of Life Insurance Offices did not carry with them complete conviction against drinking in moderation. He could not agree with those speakers who would never give alcohol in cases of *delirium tremens*. He had frequently had practical evidence of its utility in such cases, and to assist the action of appropriate sedatives he would without any hesitation prescribe it again. Mr. Dacre joined issue with Mr. Ewens in not giving alcohol to a patient during illness who was addicted to intemperance. He considered that such a man would at such times require more than any other a due allowance of alcoholic stimulant. In conclusion, he thanked the members for the kindly way in which his paper had been received, and for the lengthy discussion which had followed.

---

*February 12th*, 1896.

Dr. A. J. Harrison in the Chair.

Dr. Shingleton Smith showed two patients: (1) A boy, aged 7, who was admitted to the Bristol Royal Infirmary on November 18th, 1895, in consequence of diffuse scleroderma. He is the eldest of a family of five, all healthy. He has had measles and chicken-pox, but not scarlet fever; he has been subject to colds and has had swellings in the neck; he had what appears to have been eczema of the scalp at six months. His mother, on the day before his admission, whilst washing the child in the morning, noticed that his skin was unnaturally hard, and she is quite sure that it was not so previously. He is a healthy-looking boy, well nourished and intelligent. Weight 3 st. 10 lb. Temperature normal. The condition of skin in nearly all parts of the body is glossy, smooth, and to the touch remarkably brawny; and the face and extremities have the appearance of being swollen, although there is no œdema. The skin cannot be pinched up between two fingers. The face is immobile and manifests a lack of expression, but all the movements are performed without difficulty or discomfort. The eyelids are less tense than other parts, and can be opened and closed easily. The mouth can be opened widely, and the mucous membrane of cheeks and tongue is affected as the skin, but there is no difficulty in protrusion and other movements of the tongue. The limbs are all affected, and in all parts except the palms of the hands and the soles of the feet. At the flexures of the joints there is less induration than on the extensor surfaces, and the thighs are less brawny than the legs. The chest-wall is affected, but the movements of respiration are not hampered. There is no abnormal dryness of skin, no scaliness, pigmentation, or scars. There is no trace of albumen in the urine, and there are no indications of congenital syphilis. The thyroid appears to be normal. The condition does not produce any disability, and there are no indications of any complication like Raynaud's disease. The one thing present appears to be connective tissue overgrowth. Jaborandi had no effect whatever: doses of six drachms of infusion had no action on skin or mouth. Thyroid tabloids, two to four in the day, were given for four weeks with no effect. Massage has been continued for five weeks, and the boy appears to be somewhat improved, although the characteristic scleriasis is still present. (2) A man, aged 30, suffering from chronic chorea. The affection, of twenty years' duration, is one of muscular spasm of local athetoid type, the leading feature being a bilateral spasmodic torticollis, with an extraordinary degree of muscular hypertrophy, traumatic in origin, with no history of hereditary neurosis and no connection with rheumatism. (A full report of this case will be published in a future number.)—Dr. Henry Waldo drew attention to the suggestion that a case of a general scleroderma resembles a frozen corpse without the coldness. He said that the sudden onset fitted in with the generally accepted theory of the pathology of the disease; namely, an obstruction of the arteries, veins, and lymphatics, probably depending upon a vaso-motor lesion high up. He considered that the case shown had very much improved since he first saw it, about three months ago.—Dr. Bertram Rogers thought that the case was allied to the condition known as sclerema neonatorum, in which nodules were found subcutaneously. The condition was observed generally in children in whom a history of syphilis could be obtained, and this might account for the pathology of the disease. He asked whether any symptoms arose during the thyroid feeding, as he had given large doses to children for various complaints and had never seen any trouble arise like that he had observed in overdosing adults. He presumed that there were no symptoms of Graves's disease, though hypersecretion of the thyroid gland was now stated to be one of the causes of the disease.—Dr. Michell Clarke considered the second of the two very interesting cases shown by Dr. Shingleton Smith to be an example of the severer form of spasmodic torticollis, in which the spasm commencing in the neck had spread to the arms and shoulders. The history of onset, mode of extension to neighbouring muscles, character of the spasm, associated action of occipito-frontalis accompanying spasm of deep muscles of back of neck, were in favour of this view; as was also the absence of any sign of organic disease of the central nervous system. Chorea he considered excluded by the history and characters of the disease, and absence of family or personal history of rheumatism, or sign of cardiac disease. The rarer forms of chorea could be similarly excluded. Dr. Clarke made some remarks on two similar cases in men of 48 and 67 years of age, whom he had had under his care, and on the intractability of this severe form of the disease to treatment.—Dr. SMITH, in reply, observed that he was quite willing to accept the suggestion that the case is really one of long-standing bilateral spasmodic torticollis, with excessive overaction and hypertrophy, and suggested that division of the spinal accessory nerves might be of benefit.

Dr. MICHELL CLARKE showed a case of tabes dorsalis in a man, aged 21, the subject of hereditary syphilis. He had never acquired syphilis. The symptoms were of eighteen months' duration: the chief were Argyll-Robertson pupil, loss of knee-jerks and plantar reflexes, static and motor ataxia in legs, lightning pains and some defect of sensation in legs. Dr. Clarke also showed a man, aged 39, suffering from fibroid disease of left lung, who had been ill four years. Symptoms had greatly improved under intra-laryngeal injections of creasote and olive oil. He also mentioned the case of a girl, aged 13, with a similar condition in the right lung, who had derived great benefit from the treatment.

Dr. WALTER SWAYNE described: (1) A case of uterus bicornis in an unmarried patient, aged 18, who was suffering from profuse leucorrhœal discharge and dysmenorrhœa. She was a well-developed girl, but inclined to anæmia. There was the scar of an operation wound visible on abdomen. She had undergone abdominal section and ventrofixation of the retroflexed uterus. An American surgeon had found the uterus bicornuate, and sutured the larger horn to the abdominal wall. The condition was one of great importance in relation to menstruation with possible obstruction of one horn, and in pregnancy when rupture of the horn might occur. The uterus was found to be bicornuate and anteflexed. The left cornu was much the larger and in contact with abdominal wall, the right cornu apparently springing from it and pointing in a transverse direction. A Fallopian tube was felt at the angle of each cornu. The ovaries could also be felt. Sound passed 2½ inches into right cornu. A purulent discharge was observed coming from a sinus just in front of cervix at junction of cervical and vaginal mucous membrane. A probe was passed into utero-sacral ligament, the sinus dilated, and chloride of zinc (10 grs. to ℥j.) applied and drainage tube inserted. The patient was well a week later. (2) Two cases of absence of uterus: (*a*) A patient, aged 25, had been married seven years. She sought advice on account of sterility, and pain in the abdomen accompanied by slight swelling recurring each month. She was a well-developed woman. The breasts and external organs were normal: the pubic hair did not extend over abdomen. The vagina was normal in width, 3½ inches long, but terminated in a *cul-de-sac*. No trace of cervix or uterine body. Both ovaries were present, and a septum (probably broad ligament) could be felt running across pelvis, and in the centre of which was a very indefinite thickening (probably undeveloped tissue representing uterus). The question of oöphorectomy to relieve her menstrual pain was put before her, but the condition did not appear to be sufficiently acute to justify operation. Her sexual relations were normal. (*b*) A patient of Mr. Greig Smith's, unmarried, aged 17. Well developed externally, but with no trace of vagina or uterus. Right ovary could be felt fairly easily, left only very indistinctly. She had been sent to Mr. Greig Smith to be operated on for vaginal atresia, but in the absence of a uterus any operation appeared unjustifiable.—Dr. AUST LAWRENCE said that during twenty-one years at the Bristol General Hospital one woman had come under notice in whom there was absence of uterus and ovaries. She had no menstrual molimen. The vagina was about half-an-inch in length. He had had under his care a case of uterus bicornis in which one os uteri was patent. On the other side there was no os. This half of the uterus became distended with menstrual secretion and caused a large tumour to form, occupying the posterior half of the pelvis and rising up into the abdomen. With antiseptic precautions this swelling was opened in the vagina. The patient did well until the tenth day, when symptoms of collapse suddenly set in and death took place. The necropsy showed that the Fallopian tube had torn away from some adhesions, and the escape of blood into the pelvis produced shock and death.

Dr. A. J. Harrison read notes of the case of a man, aged 27, a tripe-dresser, who was admitted into the Bristol General Hospital on September 30th, 1895, with symptoms and history which rather pointed to cirrhosis of the liver, but which after death proved to be malignant (alveolar sarcoma) disease of the transverse colon, with secondary deposits in mesenteric glands and liver. On admission the man had no jaundice; no ascites; no albuminuria; no marked cachexia; no diarrhœa; no hæmatemesis; no melæna; no distension of abdominal veins; very little pain; only occasional sickness; no pyrexia on admission; but he had an enormous liver. This enlargement increased rapidly, and on November 21st he died rather suddenly, œdema of legs and some little ascites having come on a few days before. The liver weighed 20 lbs. 6½ oz.

J. Paul Bush, *Hon. Sec.*

---

### Cardiff Medical Society.

*January 3rd*, 1896.

Dr. D. Edgar Jones, President, in the Chair.

Mr. J. Lynn Thomas showed a specimen of chondro-sarcoma of the testis and epididymis which he had removed from a man thirty-five years of age. He also exhibited a large fibroma of the ovary. The tumour weighed 5 lbs. 8 oz. It contained two large papillomatous cysts. It had been tapped five times within the last few years, and the adhesions were very extensive. The patient made a good recovery.

Dr. R. S. Stewart entered more fully into several of the points which at the last meeting he had brought forward in his paper on the increase of general paralysis of the insane in England and Wales. He discussed the view that syphilis was a prominent factor in the causation of general paralysis, and pointed out the great difficulty of obtaining trustworthy data on this point. The discussion was joined in by several members.

---

*February 7th*, 1896.

Dr. D. Edgar Jones, President, in the Chair.

Dr. Tatham Thompson exhibited a patient in whom he had performed Mules's operation for ptosis. The patient, a girl of sixteen, had suffered from congenital ptosis of one eye. The result was excellent, the lifting of the upper lid above the corneal margin being well marked, and no discomfort was complained of, the wound having healed over the silver suture.

Dr. Rhys Griffiths showed the following specimens: (1) A gum elastic catheter with stilet, which he had removed from the male bladder, where it had lain some time. (2) A Zwancke's pessary. This had been inserted by a medical man in the vagina two-and-a-half years before for prolapse of the uterus, and had never been removed. The patient came complaining of incontinence of urine, and it was found that the pessary had ulcerated into the bladder. It was removed with difficulty and found to have the wings much eroded. (3) A fœtus, in which the abdominal wall was deficient. This had caused considerable difficulty in recognising the presentation. (4) A brain from a case in which the lateral ventricles had been tapped. The patient had complained of symptoms pointing to the presence of cerebellar tumour, for the relief of which the skull was trephined. A trocar was passed into the ventricle and fluid withdrawn. Two or three hours later, signs of respiratory failure set in and death took place. On *post-mortem* examination, recent hemorrhage into the lateral ventricles was found. The tumour was a glioma, involving the corpora quadrigemina and the adjacent parts.

Mr. C. A. Griffiths read a paper on the "Surgery of Empyema." He advocated free incision of the pleural cavity combined with the resection of ribs, and exhibited a tube which he had devised for draining the pleura, and in which there was an arrangement for making it self-retaining.

D. R. Paterson, *Hon. Sec.*

---

### Devon and Exeter Medico-Chirurgical Society.

*February 21st*, 1896.

Mr. J. D. Harris, President, in the Chair.

Dr. C. N. Lovely showed a case of congenital deformity in a child aged about three. In the left hand the index and middle, and also the ring and little, fingers were webbed together. The thumb was normal. In the left leg the tibia and fibula were normal; there was a rudimentary astragalus, but all the rest of the foot was absent. On the right side there was an intra-uterine amputation of the right arm above the elbow, and also of the right thigh above the knee. The child was intelligent. It walks on the left knee and right stump. There was a history of the mother having been frightened by an Ostend or skinned rabbit, and Dr. Lovely suggested the theory of maternal impression.

Dr. R. V. Solly showed a case of multiple lipomata.

The President showed a specimen of dermoid cyst of ovary with a tooth *in situ*.

Mr. A. C. Roper showed a specimen of carcinoma of the sigmoid flexure.

Mr. A. C. Roper read a paper on "Perineal Pressure in Cycling." He first mentioned some anatomical points bearing on the question, and he then described how he himself had suffered from retention of urine and cystitis from this perineal pressure. He then exhibited various kinds of saddles, and discussed their individual advantages and disadvantages, and finally he showed a saddle he himself had designed. Saddles, he said, might be divided into seats and saddles. The seats had no peaks extending between the thighs, and consisted of cushions, pneumatic or otherwise, with depressions for the tubera ischii. There could be no perineal pressure, but it was extremely difficult to remain in the seat while riding. The saddles were either treed or suspended, and he recommended the suspension saddle. It should be horizontal, with a perineal slit in it, and must be capable of a slight tilt backwards if necessary.

R. V. Solly, *Hon. Sec.*

### Torquay Medical Society.

*December 18th*, 1895.

Mr. P. Q. Karkeek in the Chair.

Mr. C. H. Wade read a communication on "Medical Defence." After the discussion which followed, a resolution was put and carried unanimously to the effect: "That it is desirable in the interests of general practitioners that the British Medical Association should include medical defence as a branch of its organisation."

Mr. G. Young Eales showed: (1) A periosteal tumour of the shaft of the femur, associated with effusion of fluid into the knee and absence of knee-jerk on both sides. (2) A case of lymphadenoma.

---

*February 19th*, 1896.

Dr. Reginald Pollard, President, in the Chair.

Mr. G. Young Eales showed an unusual case of epithelioma of the mouth, and also read a communication on "Influenza."

G. Young Eales, *Hon. Sec.*

## Figures and Tables

**Figure f1:**
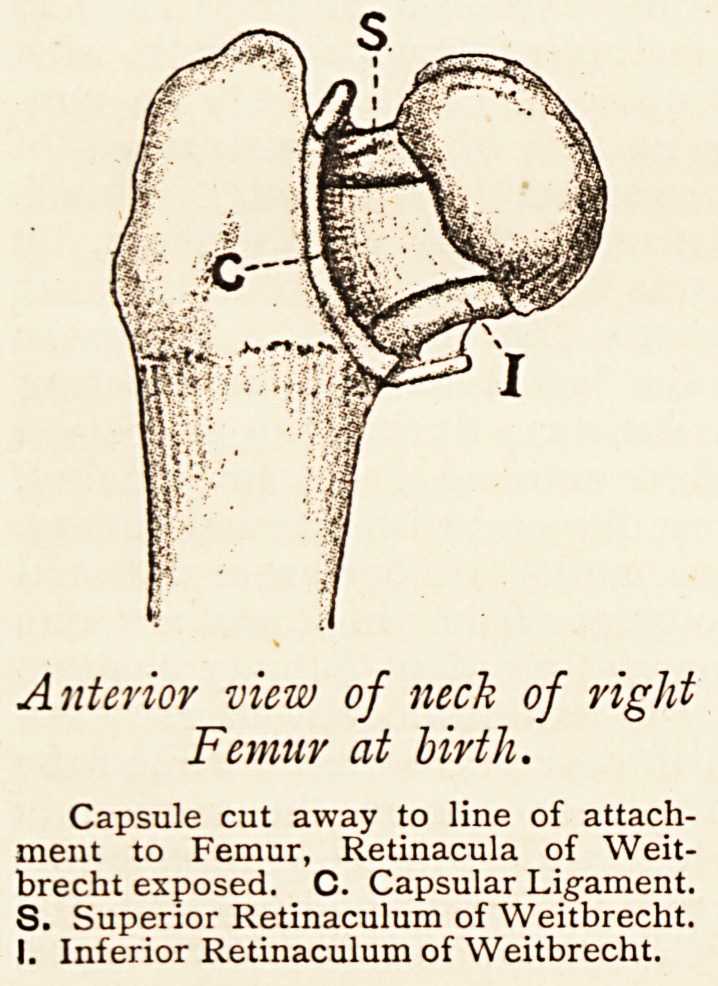


**Figure f2:**